# Electrochemical Determination of Glycoalkaloids Using a Carbon Nanotubes-Phenylboronic Acid Modified Glassy Carbon Electrode

**DOI:** 10.3390/s131216234

**Published:** 2013-11-27

**Authors:** Huiying Wang, Mingyue Liu, Xinxi Hu, Mei Li, Xingyao Xiong

**Affiliations:** 1 College of Horticulture and Landscape, Hunan Agriculture University, Changsha 410128, China; E-Mails: iris.wang@foxmail.com (H.W.); liumingyue58@126.com (M.L.); huxinxi163@163.com (X.H.); Meili_hyvri@126.com (M.L.); 2 The Institute of Vegetables and Flowers, Chinese Academy of Agricultural Sciences, Beijing 100081, China

**Keywords:** carbon nanotubes, phenylboronic acid, glycoalkaloids, sensor

## Abstract

A versatile strategy for electrochemical determination of glycoalkaloids (GAs) was developed by using a carbon nanotubes-phenylboronic acid (CNTs-PBA) modified glassy carbon electrode. PBA reacts with α-solanine and α-chaconine to form a cyclic ester, which could be utilized to detect GAs. This method allowed GA detection from 1 μM to 28 μM and the detection limit was 0.3 μM. Affinity interaction of GAs and immobilized PBA caused an essential change of the peak current. The CNT-PBA modified electrodes were sensitive for detection of GAs, and the peak current values were in quite good agreement with those measured by the sensors.

## 1. Introduction

**Figure 1.**
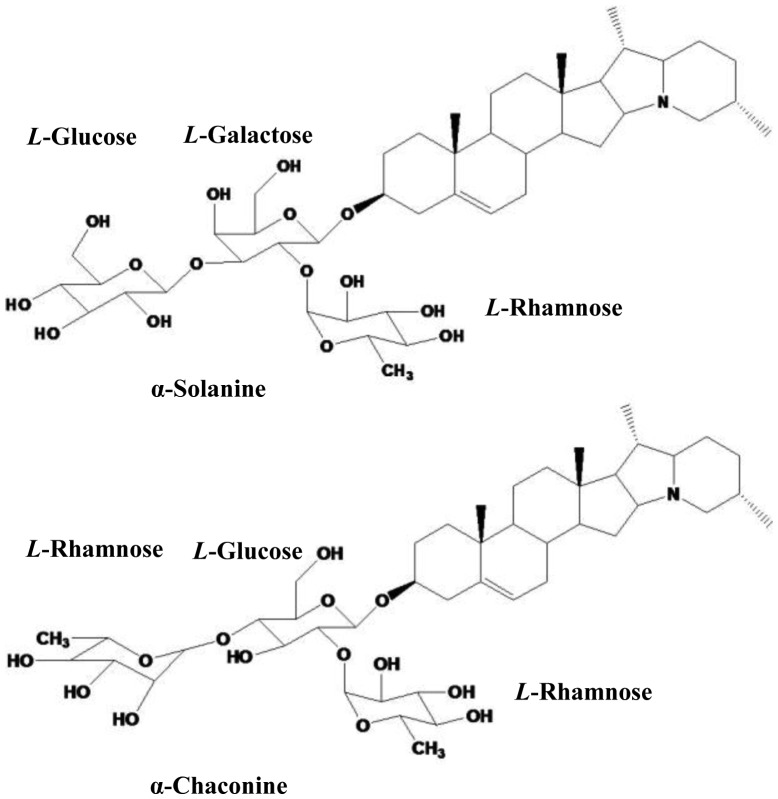
Structures of α-solanine and α-chaconine.

Glycoalkaloids (GAs) have been found in several vegetables and fruits, particularly in potatoes. The potato GAs are important components of plant resistance against pests and pathogens, but can be toxic to humans at higher concentrations [1]. The major components of potato GAs are α-solanine and α-chaconine (Figure 1), accounting for approximately 95% of the total GAs in potatoes [2–4]. The sub-cytotoxic concentrations of potato GAs and potato peel extracts possess anti-inflammatory effects *in vitro* and with further investigation may be useful in the prevention of anti-inflammatory diseases [5]. As food ingredients, the anticarcinogenic effects of potato GAs against human cervical, liver, lymphoma, and stomach cancer cells have been studied [6]. The molecular targets underlying the cell death-inducing activity of the GAs also have been examined [7]. Several methods have been used to detect GAs, such as High Performance Liquid Chromatography (HPLC) and Liquid Chromatography-Mass Spectrometry (LC-MS) [8,9]. Some enzyme-based biosensors were developed for detection of GAs, such as butyrylcholinesterase biosensors based on pHISFETs and non-Faradaic impedimetric biosensors [10,11]. Among them, the most widely used one is HPLC, a chromatographic technique for separation, identification and quantification of compounds in a mixture. It is preferable in detection of a large number of specimens due to its advantages of high-throughput and high sensitivity, however, in the case of small numbers of specimens, the operation of the HPLC instrumentation becomes time-consuming, complex and costly. HPLC also employs sometimes toxic solvents such as acetonitrile and methanol in the operation and may be toxic to humans after long-term exposure. The recorded anticarcinogenic effects of GAs show that, in the testing of isolated pure α-chaconine and α-solanine, artificial mixtures of the two GAs and total GAs compounds from fresh potatoes, all samples reduced the numbers of the following human cell lines: cervical (HeLa), liver (HepG2), lymphoma (U937), stomach (AGS and KATO III) cancer cells and normal liver (Chang) cells. However, the effects of the GAs were concentration dependent in the range of 0.117–11.7 μM [12]. The results indicate that quantifying the GAs performs more practical role than simply identifying its components. If the GAs can be directly detected by some systems such as a GAs meter or a GAs test strip, the anti-inflammatory research of GAs will be improved, and will be helpful for the related research on the anticarcinogenic potential of food ingredients.

Carbon nanotubes (CNTs), which can be divided into multi-walled carbon nanotubes (MWNTs) and single-walled carbon nanotubes (SWNTs) [13], are of great interest for the fabrication of new classes of advanced materials. Their unique electronic properties show that CNTs have the ability to promote electron transfer reactions when used as an electrode modifier in chemical reactions [14–16]. These properties make them extremely attractive for fabricating sensors [17–20]. Phenylboronic acid (PBA)-immobilized CNTs modified glassy carbon (GC) electrodes have shown improved sensitivity and selectivity towards analytes.

PBA and its derivatives could be used as recognition elements for the construction of electrochemical and optical glucose sensors [21]. PBA is a synthetic molecule capable of reversibly binding with 1.2- or 1.3-diols, hallmark structures for a majority of glycan constituent saccharides [22]. PBA forms a negatively charged species in alkaline media as a result of OH^−^ addition from solution. The anionic form of PBA is characterized by an electron-rich sp^3^ boron atom with tetrahedral geometry, whereas the non-ionic form is an electron-deficient Lewis acid with a sp^2^-hybridized boron atom. In the presence of sugar, PBA binds to the sugar to form a cyclic ester. Many papers have reported the progress in the design of PBA-based electrochemical and optical sensors for the detection of glucose and other sugars [21]. However, PBA modified electrode used for the detection of GAs (α-solanine and α-chaconine) has not been reported to date.

The main objective of this study is to propose a theoretical research support for the development of the simple, fast way to determine GAs. A new sensor based on CNTs-PBA modified GC electrode was fabricated for the determination. The sensitivity and stability of the sensor were investigated. For comparison, HPLC was employed as a reference to measure the same samples which were total GAs isolated from fresh potato samples.

## 2. Material and Methods

### 2.1. Reagents

All solutions were prepared from analytical grade reagents and MilliPore MilliQ ultrapure water (resistivity 18.2 MΩ·cm). Crystal α-solanine and α-chaconine were purchased from J&K (Beijing, China). 3-Aminophenylboronic acid was purchased from Aldrich Chemical Co. (St. Louis, MO, USA). N-hydroxysuccinimide (NHS) and 1-ethyl-3-(3-dimethylamino-propyl) carbodiimide hydrochloride (EDC) were obtained from Shanghai Medpep Co., Ltd. (Shanghai, China). MWNTs (95%, 20–60 nm) was purchased from Shenzhen Nanotech. Port. Co. Ltd. (Shenzhen, China). Further purification was accomplished by sonicating CNTs in a mixture of concentrated sulfuric acid-nitric acid (3:1, v/v) for about 12 h. The treated CNTs were filtered and washed with ultrapure water, and then dried in a vacuum at 60 °C. The black powders were sonicated in ultrapure water for about 2 h with a concentration of 1 mg·mL^−1^. The experiments were carried out at room temperature.

### 2.2. Materials

Five different fertilizer levels of Favorita, a commercial potato variety, were prepared as the simples for investigation. Five kilograms of each tuber sample were randomly collected from the fields after maturity. The samples were fresh potatoes without storage before making the experiments. All tubers were washed and dried before being peeled. Five grams of the liquid material, which was obtained after grinding the samples in an electric grinder, was used for GAs extraction. As an extraction solvent, methanol-formic acid (1:1, v/v) was used. Fifty millilitres of extraction solvent and 5 g of liquid material were sealed in an iodine number flask. Then the samples were incubated in an ultrasonic cleaner (10,000 Hz) for 1 h to obtain a homogenate. The samples were centrifuged at 10,000 rpm for 10 min. Before use, the solvents were filtered through a 0.45 μM membrane filter (Millipore, Bedford, MA, USA). All the processes were carried out at room temperature.

### 2.3. Apparatus

Electrochemical measurements were carried out on a CHI660D electrochemical workstation (Chenhua Instrument, Shanghai, China) with a conventional three-electrode cell. A glassy carbon (GC) electrode was used as the working electrode. A saturated calomel electrode (SCE) and a platinum column were used as the reference and counterelectrodes, respectively. It should be noted that, all the potentials in this paper were in respect to SCE, unless otherwise stated. The electrochemical measurements were carried in Fe(CN)_6_^3−/4−^ solution (2.5 mM, pH 7.2). A Bomem MB 100 FT-IR spectrometer (ABB Bomem, QC, Canada) was used to characterize the CNT-PBA and the CNT-PBA/GAs.

The GAs (α-solanine and α-chaconine) were separated and quantified using an Agilent Technologies (Santa Clara, CA, USA) 1260 Infinity system equipped with a UV-Vis detector. A reverse phase Agela Technologies (Santa Clara, CA, USA) Venusil XBP C18 (5 μm, 100 Å, 4.6 × 250 mm i.d.) column was used for separation of glycoalkaloids. 0.4% phosphoric acid in water and acetonitrile (1:1, v/v) were used as mobile phase with 6.5 pH value at 0.8 mL min^−1^ flow rate. The detection was done at 210 nm with the UV detector by injecting 20 μL of prepared sample after adjustment of column temperature at 25 °C.

The glassy carbon electrode (GC electrode, 3 mm in diameter) was polished to a mirror-like surface with 0.3 μm and 0.05 μm alumina slurry and then washed ultrasonically in water and ethanol for several minutes. The cleaned GC electrode was dried with high-purity nitrogen stream. Ten microliters of CNTs suspension was dropped on the surface of the pretreated GC electrode and dried under an infrared lamp to form the CNTs modified electrode (CNTs/GC). The CNTs/GC was immersed into dimethylsulfoxide (0.1 M) containing EDC/NHS (0.18 M each) for about 3 h and then washed thoroughly with ultrapure water, while the CNTs/GC was subsequently immersed into 3-aminophenylboronic acid (50 mM) for about 3 h, after that washed thoroughly with ultrapure water and dried at the room temperature to get the PBA modified CNTs/GC (PBA/CNTs/GC). Ten microliters of GAs (α-solanine) solution was dropped on the surface of the PBA/CNTs/GC. Finally, the CNTs-PBA/GAs modified electrode could be obtained after the solution was dried.

## 3. Results and Discussion

**Scheme 1.**
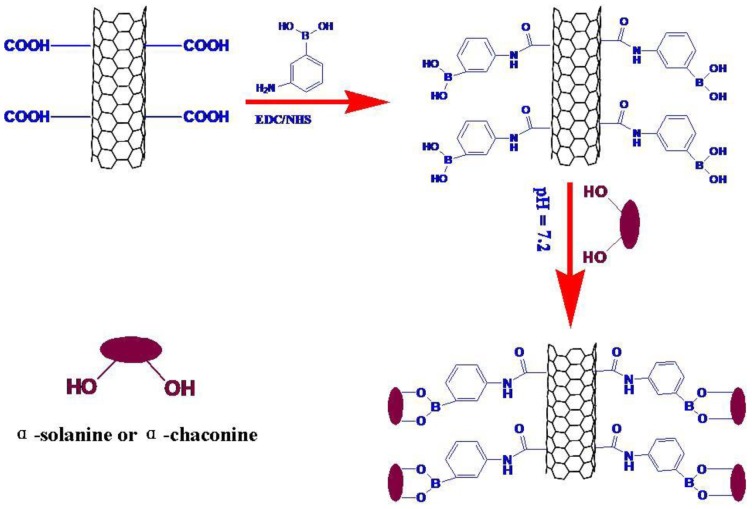
The PBA was immobilized to the surface of CNTs modified GC electrode through a carboxyl-amino reaction.

Phenylboronic acid immobilized on GC electrode can be bound with sugar and its derivatives specifically [23–27], which is commonly used for the accumulation of sugar and its derivatives. In this research, the PBA was immobilized to the surface of CNTs-modified GC electrode through a carboxyl-amino reaction as shown in Scheme 1. The CNTs were treated with mixed concentrated acid solution for 12 h, H_2_SO_4_-HNO_3_ = 3:1 (v:v). Meanwhile, a high density of carboxyl groups was generated. PBA was introduced through the reaction of amine-terminated PBA with the carboxyl groups of the CNTs using EDC/NHS as the coupling agent. The CNTs-PBA modified electrode was obtained on the GC electrode to form a sensor for the recognition of α-solanine or GAs based on the sugar–boronic acid interaction. Since PBA could form a negatively charged species in alkaline media as a result of OH^−^ addition from solution, the electrostatic repulsion would increase between the surface of CNTs-PBA/GC electrode and the Fe(CN)_6_^3−/4−^ in solution. Due to the adjacent hydroxy groups in α-solanine as well as α-chaconine, either of them could bind to PBA to form a cyclic ester (PBA was reported to react with 1,2-diols to reversibly form cyclic boronate esters [28–31]; α-chaconine and α-chaconine contain 1,2-diol structures which was used for binding with PBA to form PBA pinacol esters). In order to detect the concentration of GAs, the CNTs-PBA-modified GC electrode for determination of the concentration of α-solanine and α-chaconine was developed. In this research, α-solanine was used as test object. In the presence of α-solanine, PBA binds to it to form a cyclic ester. The cyclic ester decreases the accumulation of negative charges in the alkaline media. The electrostatic repulsion between the surface of CNTs-PBA/GAs/GC electrode and the Fe(CN)_6_^3−/4−^ in solution decreases. According to the mechanism, a sensor determination of α-solanine through changing electrochemical measurement signals was thus obtained.

### 3.1. FTIR Analysis of the Modified Electrode

**Figure 2.**
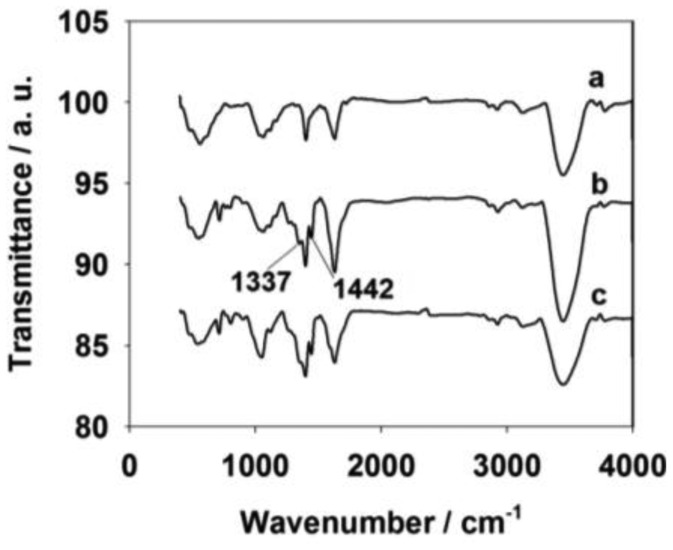
FT-IR spectrometry: (**a**) CNTs; (**b**) CNTs-PBA; (**c**) CNTs-PBA/GAs.

The formation of CNTs, CNTs-PBA and CNTs-PBA/GAs was confirmed by Fourier Transform InfraRed (FT-IR) spectrometry (Figure 2). The FT-IR spectrum of the CNTs (curve a) shows characteristic carboxyl group peaks, including the C=O stretching peak at 1,651 cm^−1^, the O–H stretching peak at 3,457 cm^−1^, the coupled C–O stretching peak at 1,030 cm^−1^ and the O–H bending vibrations at 1,416 cm^−1^[32]. These peaks revealed the success of CNT formation by acid treatment For the CNTs-PBA (curve b), peaks related to the PBA appeared, including a B–O stretching peak at 1,337 cm^−1^ and a B-C stretching peak at 1,442 cm^−1^[33,34]. For curve c, after adding α-solanine onto the CNTs-PBA/GC electrode, the coupled C–O stretching peak at 1,030 cm^−1^, and the O–H bending vibrations at 1,416 cm^−1^ became larger than that of curves a and b. To investigate the extra error of undesired interference source, the real samples test was followed. HPLC was used as a reference to measure the same samples. The relative error confirmed that there was almost no interference. The change of curve c was thought to be the existence of the PBA and PBA-α-solanine pinacol ester in the CNTs-PBA/GAS material.

### 3.2. Electrochemical Characterization of the Modified Electrode

**Figure 3.**
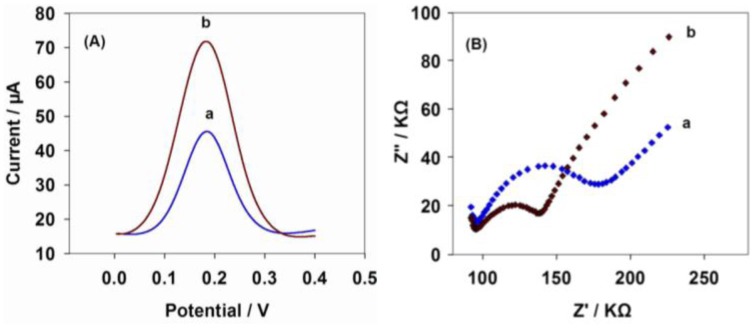
Electrochemical characterization of CNTs-PBA/GC electrode. (**A**) DPV response of the CNTs-PBA/GC electrode (curve a) and the CNTs-PBA/GAs/GC electrode (curve b); (**B**) Electrochemical impedance spectroscopy (EIS) for the CNTs-PBA/GC electrode (curve a) and the CNTs-PBA/GAs/GC electrode (curve b). The concentration of GAs (α-solanine) is 25 mM in Fe(CN)_6_^3−/4−^ (pH 7.2, 2.5 mM).

In the next study of differential pulse voltammetry (DPV) response, the α-solanine was still used as the representative for detecting GAs. α-Solanine presents the typical DPV response with the CNTs-PBA/GC electrode (curve a) and the CNTs-PBA/GAs/GC electrode (curve b, the concentration of GAs (α-solanine) is 25 mM) in Fe(CN)_6_^3−/4−^ (pH 7.2, 2.5 mM). From Figure 3A, it is noted that a remarkable oxidation peak can be observed at 0.18 V on the CNTs-PBA/GAs/GC electrode, while very small reduction peak appears on the CNTs-PBA/GC electrode. After α-solanine was modified on the CNTs-PBA/GC electrode, the increase of the reduction peak should originate from the formation of PBA pinacol ester. The negative charges of PBA pinacol ester was weaker than that of PBA, thus decreasing the electrical repellence between the modified electrode and [Fe(CN)_6_]^3−/4−^.

Figure 3B shows the typical electrochemical impedance spectroscopy (EIS) for the CNTs-PBA/GC electrode and the CNTs-PBA/GAs/GC electrode, which were recorded at the open-circuit potential in Fe(CN)_6_^3−/4−^ (pH 7.2, 2.5 mM). The change tendency of EIS is also in accordance with Figure 3A. In the presence of α-solanine, there is a large decreasing trend in the AC impedance. The possible reason was thought to be caused by the cyclic ester formed by PBA molecules binding to the α-solanine, which decreased the electrostatic repulsion between the redox probe and the electrode surface. The results shown in Figure 3 imply that CNTs-PBA/GAs/GC electrode has been successfully prepared according to Scheme 1, and it could be used to electrochemically detect GAs.

### 3.3. The Optimization of the Experiment Conditions

Five levels of CNTs were investigated by DPV in Fe(CN)_6_^3−/4−^ (pH 7.2, 2.5 mM). The concentrations were 0.0, 0.5, 1.0, 1.5 and 2.0 mg·mL^−1^. It was noted that the peak currents increased with the increasing of the concentration of CNTs when the concentration of CNTs was lower than 1.0 mg·mL^−1^. However, the variation trend of the currents was almost the same when the concentration of CNTs became larger than 1.0 mg·mL^−1^, which means that the optimum CNTs amount is 1.0 mg·mL^−1^.

Meanwhile, five concentrations of PBA were investigated by DPV. They were 15, 20, 25, 30 and 35 mM. The data obtained showed that the peak currents decreased with the increasing of the concentration of PBA but almost stayed at the same value when the concentration of PBA was higher than 25 mM. It indicated that the 25 mM was the optimum PBA amount.

### 3.4. DPV Responses of the Modified Electrode

**Figure 4.**
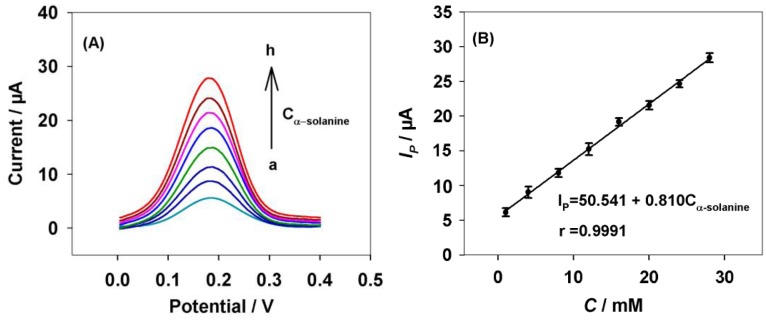
DPV responses of α-solanine at the CNTs-PBA/GC electrode. (**A**) DPV responses on the CNTs-PBA/GC electrode after incubated with different concentration of α-solanine, recorded in PBS (0.1 M, pH 7.2) containing [Fe(CN)_6_]^3−/4−^ (2.5 mM). (a) 1.0 μM, (b) 4.0 μM, (c) 8.0 μM, (d) 12.0 μM, (e) 16.0 μM, (f) 20.0 μM, (g) 24.0 μM, (h) 28.0 μM; (**B**) Plot of the peak current *versus* the concentrations of α-solanine.

Under the optimized experiment conditions, the DPV responses for different concentrations of α-solanine are shown in Figure 4.

It was noted that the peak current of α-solanine increased linearly with the increase of the α-solanine concentration from 1 to 28 μM (Figure 4B). The linear range was wider compared to using the impedance probe method [11]. The corresponding linear function was I*_P_* (μA) = 50.541 + 0.810 C_α-solanine_ (mM) with a correlation coefficient of R = 0.9991. On the other hand, the detection limit was 0.3 μM (S/N = 3). After a month, the sensor retained 91% of its initial response to α-solanine. To confirm the fabrication reproducibility, 12 electrodes were constructed individually for comparison. The experimental results showed that the mean relative standard deviation was 8% for the current determinations, which indicated that the sensor had a good stability and reproducibility.

### 3.5. Determination of GAs Isolated from Potato Samples

The modified electrode was evaluated by detecting the GA samples isolated from fresh potatoes (both the α-solanine and the α-chaconine contain 1,2-diols, hallmark structures for a majority of glycan constituent saccharides, that reversibly bind with PBA; the total amount of α-solanine and α-chaconine could be detected by the modified electrode). Definite amounts of the same potato samples were added onto the surface of the CNTs-PBA modified electrode to form the CNTs-PBA/GAs modified electrode. The results of the five samples were 4.54, 5.31, 5.62, 6.41 and 6.98 μM.

The same GAs samples were detected by HPLC and utilized to demonstrate the practical usage of the fabricated GAs sensor. The analysis of the real potatoes samples was performed in 10-minute intervals during the separation. The concentration of GAs in real potato samples was determined by HPLC (n = 3). The concentration of α-solanine and α-chaconine in five samples were determined. The α-solanine and α-chaconine account for approximately 95% of the total GAs in potatoes [2–4]. Thus, potato GAs determination could be achieved by detecting α-solanine and α-chaconine. The GAs concentrations of the five samples were 4.84, 5.50, 5.67, 6.61 and 7.11 μM.

The relative error between the results obtained by HPLC and the results detected by CNTs-PBA/GAs/GC electrode were: 6.51%, 3.59%, 1.01%, 3.25% and 1.91%. The mean error (3.25%) showed a good agreement in all samples. The results indicated that the sensitivity of the sensor was satisfactory.

## 4. Conclusions

In this paper, a CNTs-PBA-modified GC electrode has been used for the first time for the electrochemical determination of GAs. By reacting PBA with the adjacent hydroxy groups of α-solanine or α-chaconine to form a cyclic ester, the GAs could be detected with a simple preparation procedure. The peak current was observed to increase linearly with respect to the α-solanine concentration under the optimized experimental conditions. The linear correlation coefficient R of the linear function is almost equal to 1, which indicates that the sensor has good linearity. It was also confirmed that the sensor showed a good stability and reproducibility, even after 30 days. Real potato samples have been detected by the fabricated sensor in comparison with HPLC. The results indicated that the sensor could detect not only pure α-solanine, but also total GAs compounds in fresh potatoes. Compared with the HPLC, this method has many advantages, such as fast response, cheap instrumentation, low cost and simple operation, while its disadvantage is that with HPLC high sample throughput is possible when determining large number of specimens. It is expected that the sensor can not only promote GA research and the study of their anti-inflammatory and anticarcinogenic mechanisms but also have potential application in directly detecting GA systems. Further theoretical research for development of a simpler and faster way to determine GAs will be carried out as future work.
